# Maximizing the benefits of improved cookstoves: moving from acquisition to correct and consistent use

**DOI:** 10.9745/GHSP-D-14-00060

**Published:** 2014-07-22

**Authors:** Anita Shankar, Michael Johnson, Ethan Kay, Raj Pannu, Theresa Beltramo, Elisa Derby, Stephen Harrell, Curt Davis, Helen Petach

**Affiliations:** a Johns Hopkins Bloomberg School of Public Health, Center for Global Clean Air, Baltimore, MD, USA; b Berkeley Air Monitoring Group, Berkeley, CA, USA; c BioLite, New York, NY, USA; d Emergence, Inc, New York, NY, USA; e Impact Carbon, San Francisco, CA, USA; f Winrock International, Arlington, VA, USA; g University of Delaware, Newark, DE, USA; h United States Agency for International Development, Washington, DC, USA

## Abstract

The adoption of clean cooking technologies goes beyond mere product acquisition and requires attention to issues of cooking traditions, user engagement, gender dynamics, culture, and religion to effect correct and consistent use.

## INTRODUCTION

The goal of this article is to put forth the most critical considerations regarding cookstove adoption that were identified at a meeting of the Working Group to Address Increasing Adoption of Improved Cookstoves. The meeting was hosted by the environmental health project WASHplus (funded by the United States Agency for International Development [USAID]) and the research project Translating Research into Action (TRAction) in Washington, DC, in October 2013. In this article, we use the term improved cookstoves (ICS) to mean those that demonstrate more efficient fuel use and more reductions in the emissions of carbon monoxide and particulate matter than traditional biomass stoves. At a time when new ICS programs are being created and implemented, it is important to disseminate the latest knowledge about effective ICS adoption and use.

There are numerous examples in the developing world of products whose potential benefits far outweigh their costs but are not readily adopted. As there is no commonly accepted definition for adoption of a technology, we loosely define it as the acquisition and substantive use of a technology by the user. Some products known to suffer from this adoption puzzle include insecticide-treated bed nets, safe-water products, toilets, and ICS. Of these, ICS adoption faces some of the greatest challenges as less than 30% of biomass stove users globally cook with some form of an improved cookstove.^1^ Furthermore, access to high-efficiency, low-emission, low-cost stoves, while expanding, is still limited. Importantly, in households with an ICS, there is often incorrect, inconsistent, and non-exclusive use, a fact that can curtail the benefits to be gained.

At the household level, the benefits of ICS may include reducing the time, money, and labor required for acquiring fuel. Environmental benefits may include reductions in anthropogenic climate change and deforestation. The stoves may also have the potential to improve health by reducing exposure to household air pollution (HAP) for cooks and accompanying children.^2^ As 40% of the world's population (2.8 billion people) continue to cook on inefficient traditional cookstoves, there is considerable potential for clean-burning technologies to make a large global impact.^3^ Moreover, the “Global Burden of Disease Report”^4^ indicated that HAP was the fourth most significant risk factor for premature deaths worldwide, and the second in South Asia and sub-Saharan Africa. Although the evidence that ICS use leads to improved health is weak, a number of large-scale randomized trials are currently underway.

To maximize the energy-saving and potential health impacts from ICS, the stoves must first be acquired, then used correctly and consistently. Perhaps most critically, the stoves must come to displace the use of the traditional stoves. However, user demand for ICS is not yet sufficient to result in mass adoption. The challenge is thus to bring ICS adoption to scale. As this challenge is being addressed by numerous concurrent initiatives, in this article we focus on the critical aspects of building consumer demand and ensuring the correct and consistent use of ICS.

Successful ICS adoption goes beyond acquisition to ensure correct and consistent use.

## BUILDING DEMAND FOR ICS

Although ICS may be considered “adopted” once it is acquired, it will not displace traditional technology without correct and consistent use. Nonetheless, the most basic requirement for ICS adoption is acquisition, which necessitates that consumers know about and have access to ICS and are motivated to buy one. It also requires that consumers have the decision-making power and the economic resources to make the purchase.

### Financing Options

Impact Carbon, a household energy implementing organization based in San Francisco, CA, successfully increased the acquisition of a rocket-style stove in villages in and around Mbarara, Uganda, from 4.6% to 57% by using a novel sales offer that included free trials, time payments, and return options.^5^ In a related study, as part of an initiative funded by USAID/India, Abt Associates connected stove sales agents with a microfinance institution in Uttarakhand, India, to facilitate increased consumer access to purchasing capital, which resulted in more than 200 stove purchases.^6^

### Marketing Campaigns

Marketing plays a powerful role in demand creation and in the accumulation of goods in almost every society. However, marketing campaigns to promote cookstoves have yielded mixed results over the past decades, and generally adoption rates remain low. Two types of product marketing techniques are generally used in the household energy sector:

1. Carefully crafted mass marketing campaigns
2. Focused approaches to engage households deeply and consistently through locally appropriate product demonstrations and follow-up visits

In the case of mass marketing campaigns, targeted consumer engagement can be effective. Several important lessons from marketing frameworks commonly used by global private-sector advertising may be applicable to the uptake of ICS.^7^ Generally, effective marketing requires a creative strategy that is built around insights about consumers; in contrast, public health formative research is generally limited to end-user perspectives and habits. Effective advertising is rarely proscriptive. Rather, their messages engage the consumer in a simple, directional manner that piques interest and over time drives behavior change. Cookstove promoters should be confident in marketing the product based on what the consumer has identified as the most important attribute.

Experience has shown that status can play an important role in ICS adoption, as when potential customers learn the adoption choices of opinion leaders in their community.^8^ Established social networks and peer contacts may also influence perceived stove status as well as facilitate learning about stove attributes and performance. Perhaps in recognition of this, several stoves are being manufactured using bold signature color schemes and components that call attention to their perceived high level of technical sophistication. Empirical evidence of consumer patterns of Internet purchases in higher-income countries reveals that when given price options, people do not always choose the cheapest option. The same can be true in lower-income countries, particularly where status can be shown to be a driver of decision-making.^8^

There is substantial evidence that health-related messaging, while important in increasing health knowledge, does not actually increase ICS sales and adoption.^9^^–^^12^ The Shell Foundation's Room to Breathe social marketing campaign in Southern India, which used television and radio advertising, raised awareness of the risks of household air pollution from 43% to 69% in their campaign districts according to a post-campaign survey.^13^ Moreover, 83% of respondents said they would buy an “improved” stove, but only 2% actually did. In this case, traditional mass marketing raised awareness, but it did not drive new cookstove purchases.

Instead, studies are recognizing the importance of non-health motives, including cost and intra-household gender dynamics, in adoption decisions.^14^^,^^15^ But even here, the experience can be mixed. A recent experiment in rural Uganda tested the impact of marketing messages on willingness to pay for an ICS (measured as an actual purchase, not a hypothetical question). Despite locally developed marketing messages and best-practice strategies including vivid messaging and local experience with the ICS, there was no consistent increase in the willingness to pay as a result of the message, “the stove can improve health,” or the message, “the stove can save time and money.” Thus, there is a critical need to understand underlying user preferences and hidden costs beyond health in the design and delivery of ICS, specifically, how external and intra-household relations shape decisions regarding energy and technology acquisition and use.^16^

Marketing campaigns should focus on the key product attributes identified by consumers.

### Effective User Engagement

ICS is a new product category for many households. For ICS to be adopted, retailers need to engage with users directly. In a recent study of 10 stove manufacturers in India, all 10 companies identified product demonstrations as the most effective driver of stove adoption.^17^ In addition to letting customers see and use the stove, demonstrations also helped address product perception issues. For instance, in Maharashtra, India, customers routinely question whether an improved stove can cook *chapatis*, the local flatbread, as effectively as on an open fire. Manufacturers have concluded that the best way to prove the capability to cook local cuisine is to let prospective customers taste the results.

However, engagement should not stop at the point-of-sale. With any new technology, there is a user learning curve. In addition to training at the point-of-sale using formal and informal input, customers should receive regular follow-up visits until they have mastered the technology. These visits are critical to fostering correct and sustained use of the new stove. Without them, customers abandon the product and go back to using an open fire—a vexing reality faced by many public health intervention implementers.^18^ The worst outcome would be substantial numbers of poor households investing in a relatively expensive new appliance and not using it.

Retailers need to engage with users through demonstrations, training, and post-sales support.

The extent of behavior change required on the part of the user affects consumer demand. In the case of cookstoves, the behavioral shift required for ICS use is significant, particularly when compared with the behavior shift required for health programs such as vaccines or vitamin distribution. ICS use requires numerous changes on a daily basis that are often associated with a financial cost and that break with long-standing family cooking tradition. Therefore, it is important that manufacturers design products that are more consistent with local practices rather than trying to substantially change cooking practices and fuels. Finally, correct and consistent use of ICS requires that consumers are engaged as full partners in the move toward clean and efficient fuel and technologies and that they clearly understand the ICS value proposition.

### Addressing Intra-Household Gender Dynamics

In households that burn biomass fuel for cooking, women are nearly always responsible for the cooking, and along with their children, they disproportionately suffer most of the health-related consequences of HAP. By contrast, men of the household are often not home when meals are being prepared, yet they hold disproportionate control over household purchasing decisions. This non-price barrier to ICS adoption is an identified intra-household externality: male financial decision-makers do not internalize the health benefits of a new technology that accrues to their wives and children.^18^^,^^19^ In an experiment regarding the willingness-to-pay for an ICS in rural Mbarara, Uganda, 55% of households reported that women and men share joint decision-making about purchases for durable goods. However, among married households, women are willing to pay 21% to 23% less than men for an ICS, suggesting they have less spending power and less money to offer than men. In addition, when it comes to actual decision-making, the amount of power exercised by women who perceive themselves to be joint decision-makers (or who are in couples who self-identify as having shared decision-making power) is not statistically significant compared with women or couples who do not identify themselves as having shared decision-making power. Thus, even when reported as joint decision-makers with their husbands, women's power over household spending may be limited. As a response, efforts to increase willingness to pay for ICS may be more successful by designing and disseminating cookstoves with features valued more highly by men, without sacrificing the features valued by women so that they consistently use it.^20^

The Shell Foundation's aforementioned Room to Breathe campaign concluded that “94% of households said buying a stove was a joint decision between man and wife, which means social marketing must reach both audiences.” This conclusion was corroborated by a study of the First Energy Oorja stove in rural Maharashtra, which concluded that the third most common explanation for not purchasing a clean stove in Maharashtra, India, after household income and family size, was “husband not interested.”^18^ To address this lack of interest, some recent stove designs have included electricity generation to charge mobile phones.

Intra-household factors that influence decision-making can also be seen at various levels of the ICS value chain. For example, the extent to which women are involved in enterprises and programs that provide modern energy and technologies depends on their bargaining power and control over assets and resources. Growing evidence shows that uptake will be limited unless women gain more say in household purchases and access to credit. As we move toward expanding acquisition globally, it will be critical to recognize the challenges of gender-related dynamics and to find opportunities to engage women more effectively across the value chain.

### Engaging Women Across the Value Chain

From a public health perspective, women are central to improving health for themselves and their families. In general, when women have greater control over the use of household income, expenditures tend to be more focused on meeting the basic needs of the family and of the children.^21^ Including women in all aspects of energy programming could yield positive benefits for themselves and their families. Women are one of the fastest growing cohorts of entrepreneurs in many developing countries,^22^ and leveraging their strengths offers an opportunity for the energy sector. In a study of female entrepreneurs globally, researchers found that women are more likely to start businesses with both social and economic goals, or hybrid ventures.^23^ Regarding clean-cooking solutions, women's substantial informal networks can open doors for new cooking-product businesses and provide access to consumers in hard-to-reach markets. In countries where gender disparity is high, employing women as sales agents can be a way to access untapped female markets as it is often easier for women to buy directly from other women in the community than having to go to cities or marketplaces. One example is the Al Johar Initiative created by Vodafone in 2010 that engaged all-female networks to access female markets in Qatar in hopes of overcoming cultural restrictions in movement and communication with men; the women reached 100% of their sales targets.^24^

Women are uniquely positioned to promote use of ICS. As the primary energy consumers and beneficiaries of ICS, women are well-versed in understanding the challenges of ICS adoption and continued use and are therefore integral to any consumer awareness and education campaign. Several women-focused initiatives in Africa, including ENERGIA Solar Sisters and Maasai Stoves and Solar are documenting the critical role women play in promoting the use of ICS among their peers. Women can also play central roles in microenterprise and as extension workers supporting maintenance and as leaders, networkers, and promoters for ICS in their region. Considerable challenges exist, and efforts to increase both external resources and internal agency are required.^25^ Key to moving forward will be to effectively engage women in ways that accommodate or help overcome existing constraints while building intrinsic and extrinsic supports for their successful involvement.

### Additional Cultural Considerations

Religious and cultural beliefs can also be an important consideration in ICS uptake and usage. According to many households in rural India, the open fire is not just a cooking appliance, but the spiritual center of the home.^18^ Families saw the fire in their kitchen as a domestic god, a deity, and the smoke as a link between the earth and heaven. They prayed before the stove daily, and created *rangoli*, artwork drawn around the stove to consecrate it, to make it a sacred object. The religious significance of the open fire, as an obstacle to uptake of so-called “smokeless” cookstoves, is relevant in India, sub-Saharan Africa, and Latin America. For instance, many Peruvians interpret cooking smoke as a manifestation of God's presence (personal communication with A. Laurent, co-founder of Microsol, the carbon accreditation organization, Peru, 2011). These examples of gender and cultural considerations demonstrate how critical it is that ICS programs engage with communities to understand how their products will be most likely used in households.

## STOVE USE AND BENEFITS

The extent to which the new stoves are beneficial is influenced by how correctly and consistently they are used as well as by how much they displace traditional stoves. Correct use includes both operation and maintenance requirements, and it is influenced by a host of factors, including ease of use, consumer education received at the point-of-purchase, formal and informal input and advice offered to the user, compliance with proper use instructions, and how well cooking with the stove meets consumer needs and expectations. These factors affect whether the new stove is used consistently and the extent to which the new stove displaces the traditional stove, or is used alongside other cooking technologies (is “stacked”).^26^

### Health Benefits

[Fig f01]The displacement of inefficient, polluting traditional stoves is critical to achieving health benefits. For example, based on the air quality model in the “International Workshop Agreement (IWA): Guidelines for Evaluating Cookstove Performance” (ISO 2012),^27^ a 3-stone-fire would have to be used for less than approximately 1 hour per week, and there must be zero emissions from any other source in order to stay below the World Health Organization (WHO) Annual Interim 1 Target for PM 2.5 (particulate matter 2.5 μm in diameter and smaller) in the kitchen. Put simply, for protection of health at WHO levels, users not only must use an extremely clean stove but also must use it almost exclusively. Integrated exposure-response models for PM 2.5 for heart disease, stroke, and respiratory illness provide quantitative support for standards of acceptable indoor air pollution exposures.^28^

**Figure f01:**
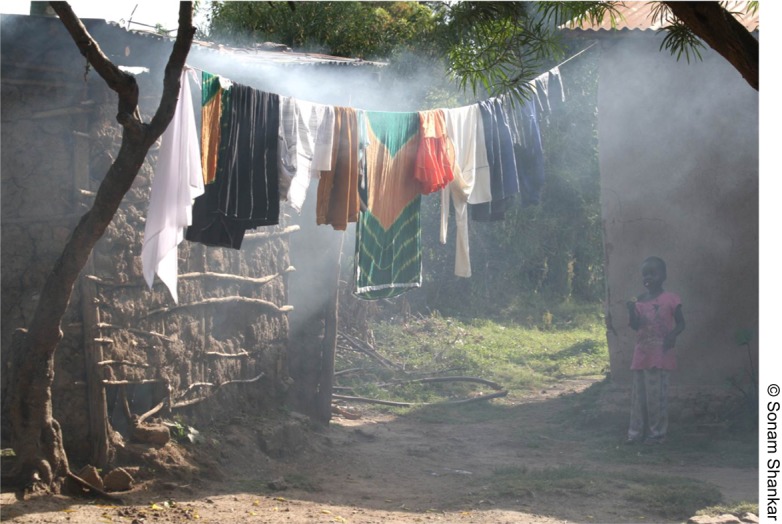
Traditional cookstoves produce high levels of ambient air pollution even outside the dwelling.

### Fuel Efficiency

From a fuel efficiency perspective, to achieve a 50% fuel savings, the most efficient cookstoves (Tier 4 for fuel efficiency as defined by the ISO International Workshop on Cookstoves^27^) must displace 70% of typical baseline stove use, or a mid-level stove in terms of efficiency (Tier 2) must be used exclusively.^29^ While currently this may appear to be unattainable in many settings, it is important to acknowledge the value of incremental progress in areas of technology, demand creation, and consumer support that is advancing us toward this goal.

### Cookstove Use Patterns

The quantification of cookstove use is possible using technologies and time-use pattern survey tools such as Stove Use Monitoring Systems (SUMS); Nexleaf Analytics Wireless Cookstove Sensors (WiCS); the SWEETSense STOVE; and direct survey tools. However, few studies have reported how consumers use cookstoves in parallel and for what tasks. Knowing which cooking tasks are responsible for the greatest emissions and fuel consumption helps stove producers ensure their designs are well-suited for those tasks, and this can inform user training efforts to strategically encourage consumers to use the ICS specifically for those tasks. Understanding the daily patterns of traditional and nontraditional cooking technologies is essential for researchers and policy makers attempting to reduce indoor air pollution and environmental degradation from inefficient cookstoves.

Stove producers must ensure their designs can handle the cooking tasks responsible for the greatest emissions and fuel consumption.

Berkeley Air Monitoring Group investigated fuel savings of the EcoChulha forced draft stove with respect to specific cooking tasks in their field study funded by the United States Environmental Protection Agency with Alpha Renewable Energy in Gujarat, India. Using the EcoChulha for only the energy intensive tasks of cooking bread and vegetables, for example, would result in 80% traditional stove displacement and 50% fuel savings.^30^ While full displacement of traditional stoves would, of course, maximize benefits, this analysis demonstrates that it can help to focus stove design and behavior change strategies on addressing the most energy-intensive tasks.

### Incremental Progress

The IWA framework recognizes the importance of incremental progress toward the larger goals of widespread adoption of cooking solutions with the highest efficiency/fuel use, emissions, and safety. As stated in the IWA,^27^ these guidelines acknowledge progress while setting aspirational goals and allow organizations and countries to select indicators and tiers based on local priorities. In addition, programs should consider whether the need for free or heavily subsidized stoves is an appropriate strategy to achieve wide-scale adoption in some settings. Ultimately, protecting health and the environment will depend on whether the household energy sector can provide cookstoves with low-pollutant emissions while also meeting consumer needs. Thus, addressing those needs will be fundamental to achieving health and environmental goals.

Addressing consumer needs is fundamental to achieving health and environmental goals.

## CONCLUSION

ICS must meet consumer needs and preferences if they are to lead to correct and consistent use and to successfully displace traditional stoves. This is also necessary for reducing household air pollution and fuel consumption, and therefore providing maximum health and environmental benefits. However, consumer needs and preferences are complex and are influenced by many contextual and social factors that require a deep understanding of culture, going beyond technology and economics. Successful ICS business models will need to be sensitive to cultural practices in both the design of the product and marketing strategies.

Key considerations that can aid in large-scale ICS adoption include:

1. Recognizing that stove adoption does not equate with stove acquisition and that long-term consistent and continuous use requires consumer buy-in and understanding of the value proposition that ICS can provide
2. Designing marketing campaigns that engage the consumer by identifying key attributes of importance to the consumer, rather than long lists of attributes that do not necessarily influence the consumer's decision
3. Ensuring effective user engagement by including demonstrations, training, and post-sales support
4. Addressing intra-household gender dynamics to enhance equity in purchasing decisions
5. Including women more effectively throughout the cookstove value chain by improving both resources and agency-based support
6. Identifying and respecting the cultural significance of cooking food
7. Understanding the actual-use scenarios of the stove (for example, boiling water for tea versus frying flat breads)

## Notes

**Peer Reviewed**

**First Published Online:** 2014 Jul 22
